# Cascading Failures in Interdependent Networks with Multiple Supply-Demand Links and Functionality Thresholds

**DOI:** 10.1038/s41598-017-14384-y

**Published:** 2017-11-08

**Authors:** M. A. Di Muro, L. D. Valdez, H. H. Aragão Rêgo, S. V. Buldyrev, H. E. Stanley, L. A. Braunstein

**Affiliations:** 1 Instituto de Investigaciones Físicas de Mar del Plata (IFIMAR)-Departamento de Física, Facultad de Ciencias Exactas y Naturales, Universidad Nacional de Mar del Plata-CONICET, Funes, 3350 (7600) Mar del Plata, Argentina; 2 Instituto de Física Enrique Gaviola, CONICET, Ciudad Universitaria, 5000 Córdoba, Argentina; 3 Facultad de Matemática, Astronomía, Física y Computación, Universidad Nacional de Córdoba, 5000 Córdoba, Argentina; 4 Departamento de Física, Instituto Federal de Educação, Ciência e Tecnologia do Maranhão, São Luís, MA 65030-005 Brazil; 5 Department of Physics, Yeshiva University, 500 West 185th Street, New York, 10033 USA; 6 Center for Polymer Studies, Boston University, Boston, Massachusetts 02215 USA

## Abstract

Various social, financial, biological and technological systems can be modeled by interdependent networks. It has been assumed that in order to remain functional, nodes in one network must receive the support from nodes belonging to different networks. So far these models have been limited to the case in which the failure propagates across networks only if the nodes lose all their supply nodes. In this paper we develop a more realistic model for two interdependent networks in which each node has its own supply threshold, i.e., they need the support of a minimum number of supply nodes to remain functional. In addition, we analyze different conditions of internal node failure due to disconnection from nodes within its own network. We show that several local internal failure conditions lead to similar nontrivial results. When there are no internal failures the model is equivalent to a bipartite system, which can be useful to model a financial market. We explore the rich behaviors of these models that include discontinuous and continuous phase transitions. Using the generating functions formalism, we analytically solve all the models in the limit of infinitely large networks and find an excellent agreement with the stochastic simulations.

## Introduction

Studying complex systems includes analyzing how the different components of a given system interact with each other and how this interaction affects the system’s global collective behavior. In recent years complex network research has been a powerful tool for examining these systems, and the initial research on isolated networks has yielded interesting results^1–3^.

A network is a graph composed of nodes that represent interacting individuals, companies, or elements of an infrastructure. Node interactions are represented by links or edges. Real-world systems rarely work in isolation and often crucially depend on one another^4–10^. Thus single-network models have been extended to more general models of interacting coupled networks, the study of which has greatly expanded our understanding of real-world complex systems. One intensive study of these “networks of networks” has focused on the propagation of failure among closely-related systems^11–26^. The great blackout of Italy in 2003 and the earthquake of Japan in 2011 were catastrophic events that demonstrated that breakdowns in power grids strongly impact other systems such as communication and transport networks, and that the failure of these networks in turn accelerates the failure of the power grid. The propagation of these “failure cascades” has received wide study in recent years^11–21,27,28^.

The simplest model of these systems consists of two interdependent networks in which nodes in one network are connected by a single bidirectional edge to nodes in a second network^11^. In this model a node is functional (i) if it belongs to the largest connected component (the “giant component”) in its own network (the internal rule of functionality) and (ii) if its counterpart in the other network is also functional (the external rule of functionality). This original model has been extended to include localized and targeted attacks^15,29–32^ and mitigation^13,25,26,33,34^ and recovery strategies^27,35^. Recently it was found that the giant component membership requirement can be replaced by a weaker requirement of belonging to a cluster of a size larger than or equal to a threshold *h* ^*^^28^. Alternatively, a heterogeneous k-core condition can be applied as an internal functionality condition in which node *i* is functional when at least ${k}_{i}^{\ast }$ nodes among its *k*_*i*_ immediate neighbors remain functional^36–40^. In this model the random failure of a critical fraction of nodes in an isolated network leads to an abrupt collapse of this network.

Although the original interdependent network model expanded our understanding of different coupled systems, the single-dependency relationship between nodes in different networks does not accurately represent what happens in real-world structures. A cascading failure model of a network of networks with multiple dependency edges has been applied to a scenario in which nodes fail only when they lose all their support nodes in the other network^14,17^, but nodes in complex real-world systems can be so fragile that the loss of a single support link can cause them to shut down. More generally, each node may require a certain minimal number of supply links connected to the nodes in the other network to remain functional. In the world-wide economic system, for example, banks and financial firms lend money to non-financial companies who must pay the amount back with interest after a stated period of time. If a single non-financial company becomes insolvent, the bank that lent money to this company will likely not fail, but if the number of companies that cannot pay back their loans is sufficiently large, the possibility of bank failure becomes real. This resembles the k-core process in a single network described above.

Here we model the process of cascading failure in a system of two interdependent networks *A* and *B* in which nodes have multiple connections or supply-demand links between networks. In the following, network *X* means either network *A* or *B*. Each node *i* in network *X* has *k*_*sX*,*i*_ supply nodes in the other network that are connected to node *i* by supply links. This node remains functional at a certain stage of the cascade of failures if the number of its functional supply nodes in the other network remains greater or equal to its supply threshold *k*_*sX*,i_^*^ ≤ *k*_*sX*,*i*_. We call this the external functionality condition. We assume that a supply threshold is predefined for each node.

In principle, this model is non-trivial even if the survival of a node in network *X* does not directly depend on the internal connectivity of network *X*. In this case our model is equivalent to cascading failures in a bipartite network composed of two sets of nodes *A* and *B* connected only by supply-demand links, i.e., these networks only have external functionality. For generality, we add to the external functionality condition an internal functionality condition that can be one of the following: a node is functional (i) when it belongs to the giant component of its network (“giant component rule”), (ii) when it belongs to a finite component of size *h* that survives with probability 1 − *q*(*h*) (“mass rule”), and (iii) when a node *i* with internal connectivity *k*_*i*_ has a number of functional neighbors greater than or equal to *k*_i_^*^ (“*k*-core rule”).

We develop a theoretical model that is solved using the formalism of generating functions. We present numerical solutions and compare them with stochastic simulations. We find that for all internal rules of functionality, increasing the ${k}_{sX}^{\ast }$ value increases system vulnerability and often causes a discontinuous transition. For the mass rule of internal functionality we find a continuous transition for some parameter values. We also study the asymptotic limit of a large number of supply links, and we find a relation between the critical threshold of initial failure and the ratio ${k}_{sX}^{\ast }$/*k*_*sX*_.

## Model

We assume that the system consists of two networks *A* and *B* with internal degree distributions *P*_*A*_(*k*) and *P*_*B*_(*k*), respectively, where *k* is the degree of a node within its own network. Each node *i* in network *A* is supplied by *k*_*sA*,*i*_ supply links from nodes in network *B*, and each node *j* in network *B* has *k*_*sB*,*j*_ demand links that act as supply links for nodes in network *A*. For simplicity we assume that the demand links in network *A* serve as supply links for nodes in network *B*, and that supply links in network *A* serve as demand links for nodes in network *B*. Thus each supply-demand link is a bidirectional link that connects a node in network *A* with a node in network *B*. If the internal degree of all nodes in networks *A* and *B* is zero, our model is equivalent to a bipartite network. We assume that the degree distribution of supply-demand links in network *A* is *P*_*sA*_(*k*) and the degree distribution of supply-demand links in network *B* is *P*_*sB*_(*k*). In principle, some nodes may not have supply links and still remain functional^13^. If this is the case, *P*_*sA*_(0) > 0.

The functionality of the nodes in both networks is related to their connections within their own network, which we call the internal rule of functionality. In addition, the state of the nodes also depends on the supply demand links that connect both networks, which we call the external rule of functionality.

We study three different internal rules of functionality:

(I) Model I (The “giant component” rule): nodes that belong to the giant component in their own network are functional.
(II) Model II (The “finite component” or “mass” rule): a finite component of size *h* remains functional with a probability 1 − *q*(*h*). If it fails, all of its nodes fail. If it survives, all of its nodes remain functional.
(III) Model III (The “k-core” rule): a node *i* with internal connectivity *k*_*i*_ remains active if the number of its functional neighbors is greater than or equal to ${k}_{i}^{\ast }$.

The external rule of functionality states that nodes in network *X* must be connected with the other network through a number of functional supply links greater than or equal to ${k}_{sX}^{\ast }$.

We call ${k}_{sX,i}^{\ast }$ the supply-demand functionality threshold of node *i*, since in principle the threshold may be different for different nodes. For conceptual simplicity, we assume that the supply thresholds are predefined for each node by random selection from a cumulative probability distribution *r*_*sX*_(*j*, *k*) = *P*(${k}_{sX}^{\ast }$ ≤ *j*|*k*_*sX*_ = *k*), where *P*(|) is the conditional probability. Alternatively, function *r*_*sX*_(*j*, *k*) can be understood as a probability that a node with *k* supply links remains functional if *j* of its *k* supply nodes in the other network remains functional.

For example, in the case of a uniform supply threshold ${k}_{sX}^{\ast }$ = *m* where *m* is a constant, the distribution *r*_*sX*_ is a step function, i.e., *r*_*sX*_(*j*, *k*) = 0 for *j* < *m* and *r*_*sX*_(*j*, *k*) = 1 for *j* ≥ *m*. Another option is linear: *r*_*sX*_(*j*, *k*) = *j*/*k*. For autonomous nodes that can survive without any functional supply nodes in the other network, ${k}_{sX}^{\ast }$ = 0. This case is included in the general scheme if we assume that *r*_*sX*_(0, *k*_*sX*_) > 0.

**Figure 1:**
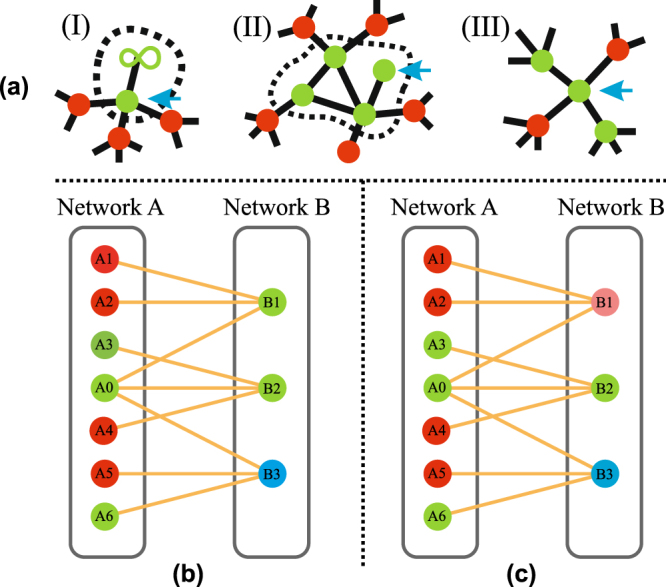
Schematic of the rules of functionality of the model. Black links represent internal connections and orange links the supplies between networks. The state of the nodes varies according to their color: functional nodes (), nodes that do not fulfill the internal rule of functionality () and nodes that fail due to the initial damage (). In addition, we have nodes that externally fail because they do not get enough supply from the other network ();. In panel (a) we show the three internal rules of functionality for a node *i* (marked with the blue arrow) to be functional: (I) it must be connected to the GC (represented by the ∞ symbol), (II) it must belong to a component of size *h* which survives with probability 1 − *q*(*h*) (in this case *k* ^*^ ≡ *k*_i_^*^), or (III) it must have a number of neighbors equal to or greater than *k* ^*^ ≡ *k*_i_^*^ (we show *k* ^*^ = 2). In panel (b) we show the external rule of functionality for *k*_s_^*^ = 1, and *k*_s_^*^ = 2 in panel (c). In these cases *P*_*sA*_(*k*) = *P*_*sB*_(*k*) = *δ*_*k*,3_, however, not all supplies are shown, nor are the internal connectivity links.

Figure 1a shows a schematic of the internal rules of functionality, and Fig. 1b,c show a schematic of the external rules of functionality. In each network green nodes are functional, i.e., they satisfy both internal and external conditions of functionality. Red nodes are affected by the initial failure, blue nodes do not satisfy internal conditions of functionality and pink nodes do not satisfy external conditions of functionality. Internal links are black, and supply links are orange. Here we use *P*_*sA*_(*k*) = *P*_*sB*_(*k*) = *δ*_*k*,3_, but for simplicity in Fig. 1b,c we omit the internal links and some of the supply-demand links in network *A*. For example, in Fig. 1b node *A*3 has two additional supply nodes from network *B* that are not shown. Figure 1b shows the case *k*_*s*,i_^*^ = 1 for all *i*. Note that since all nodes in network *B* receive supplies from functional node *A*0 they are unaffected when other nodes in network *A* fail. On the other hand, Fig. 1c shows that when *k*_*s*,i_^*^ = 2 all nodes must have two functional supply nodes from the other network to remain functional. Nodes *B*2 and *B*3 are connected to *A*0, receive supplies from functioning nodes *A*3 and *A*6, respectively, and remain active. On the other hand, because node *B*1 is only supported by node *A*0, it fails, as indicated by the pink color.

## Theoretical approach

We construct a system of two randomly connected networks in which connectivity links within each network follow degree distributions *P*_*A*_(*k*) and *P*_*B*_(*k*) and supply-demand links between the networks follow distributions *P*_*sA*_(*k*) and *P*_*sB*_(*k*). For this system we achieve a theoretical solution within the limit of a large number of nodes, *N*_*A*_ and *N*_*B*_, where *N*_*A*_ and *N*_*B*_ are the number of nodes in networks *A* and *B*, respectively. The bidirectionality of the supply-demand links requires that relation *N*_*A*_〈*k*〉_*sA*_ = *N*_*B*_〈*k*〉_*sB*_ is satisfied, where 〈*k*〉_*sA*_ and 〈*k*〉_*sB*_ are the average degrees of the supply links in networks A and B respectively.

When we randomly remove a fraction 1 − *y*_*X*_ of nodes from network *X*, the remaining fraction of active nodes *μ*_*X*_ for an isolated network *X* is determined by which internal functionality rule is followed. It can be expressed in the closed-form expression *μ*_*X*_ = *y*_*X*_ *g*_*X*_(*y*_*X*_), where *g*_*X*_(*y*_*X*_) ≤ 1 is an exacerbation factor that takes into account additional node failures triggered by the random removal of a fraction of 1 − *y*_*X*_ nodes. The explicit form of this factor is determined by the internal functionality rules of the model. The Supplementary Information presents equations for *g*_*X*_ for Rules I, II, and III (see Supplementary Information: section *Explicit form of the functionality rules*). For example, for a bipartite network *g*_*X*_(*y*_*X*_) = 1.

The cascading process begins with a random failure in network *A*. This failure causes an additional loss of nodes determined by the exacerbation factor. This event triggers a cascade in which failure is transmitted back and forth between networks *A* and *B* through the supply-demand links, and this further decreases the fraction of functional nodes. The external functionality rule states that node *i* with *k*_*s*,*i*_ supply-demand links must have ${k}_{s,i}^{\ast }$ or more nodes to remain functional, similar to k-core percolation.

External functionality failure is similar to heterogeneous k-core percolation^37^. To describe this failure due to a lack of supply between networks *A* and *B*, we introduce the functions *W*_*sA*_(*x*),*W*_*sB*_(*x*) and *Z*_*sA*_(*x*),*Z*_*sB*_(*x*), which are the k-core generating functions of the degree distribution and the excess degree distribution of the supply-demand links in networks *A* and *B*, respectively. These functions depend on the degree distributions *P*_*sA*_ and *P*_*sB*_ of supply-demand links and the distribution of the thresholds *r*_*sA*_(*j*,*k*) and *r*_*sB*_(*j*,*k*) of the supply-demand links in networks *A* and *B*,

1 $${W}_{sX}(\beta )=\sum _{k=0}^{\infty }{P}_{sX}(k)\sum _{j=0}^{k}(\begin{array}{c}k\\ j\end{array}){r}_{sX}(j,k){\beta }^{j}{\mathrm{(1}-\beta )}^{k-j}$$

and

2 $${Z}_{sX}(\beta )=\sum _{k=0}^{\infty }\frac{k{P}_{sX}(k)}{{\langle {k}_{s}\rangle }_{X}}\sum _{j=0}^{k-1}(\begin{array}{c}k-1\\ j\end{array}){r}_{sX}(j+\mathrm{1,}\,k){\beta }^{j}{\mathrm{(1}-\beta )}^{k-j-1},$$

where 〈*k*_*s*_〉_*X*_ is the average number of supply links per node in network *X*. In this context *β* is the probability that a functional node will be selected. Similar formulas were derived in ref.^41^ for a variant of the Watts opinion model^42^.

We next examine a theoretical approach to the temporal evolution of the cascading process. As explained above, initially a randomly selected fraction 1 − *p* of nodes fails in network *A*. Then the surviving fraction of nodes in network *A* in this first stage of the cascade is *μ*_*A*,1_ = *pg*_*A*_(*p*). We introduce a new parameter *f*_*B*_, which is the probability of randomly choosing a supply link that is connected to a functional node in the other network. When a node fails, all its demand links also fail. Thus *f*_*B*,1_ = *μ*_*A*,1_.

After applying the external functionality rule to network *B*, the fraction of nodes that fulfill the conditions is given by *y*_*B*,1_ = *W*_*sB*_(*f*_*B*,1_). Because there are additional disconnected nodes in network *B* given by the exacerbation factor *g*_*B*_, the number of functional nodes in network *B* at the first stage of the cascade is *μ*_*B*,1_ = *y*_*B*,1_ *g*_*B*_(*y*_*B*,1_). In the second stage of the cascade we cannot apply the same rules to obtain *μ*_*A*,2_, because *f*_*A*,2_ ≠ *μ*_*B*,1_. If, for example, a supply-demand link connects nodes *i* in *A* and *j* in *B*, then the probability that this link is active depends on how many other links belonging to nodes *i* or *j* are active. Thus the fraction of surviving links at this step is *f*_*A*,2_ = *Z*_*sB*_(*f*_*B*,1_)*g*_*B*_(*y*_*B*,1_).

Thus the recursion relations for the stages *n* > 1 are

3 $$\begin{array}{rcl}{f}_{A,n} & = & {Z}_{sB}({f}_{B,n-1})\,{g}_{B}({y}_{B,n-1});\\ {f}_{B,n} & = & p\,{Z}_{sA}({f}_{A,n})\,{g}_{A}({y}_{A,n}),\end{array}$$

where

4 $$\begin{array}{rcl}{y}_{A,n} & = & p\,{W}_{sA}({f}_{A,n});\\ {y}_{B,n} & = & {W}_{sB}({f}_{B,n})\end{array}$$

are the fractions of nodes that satisfy the external rule of functionality, i.e., *randomly* removing a fraction of 1 − *y*_*X*,*n*_ nodes leaves the same number of functional nodes as in stage *n* of the cascade. The fractions of functional nodes at stage *n* of the cascade are

5 $$\begin{array}{rcl}{\mu }_{A,n} & = & {y}_{A,n}\,{g}_{A}({y}_{A,n});\\ {\mu }_{B,n} & = & {y}_{B,n}\,{g}_{B}({y}_{B,n}\mathrm{).}\end{array}$$

The process begins with *f*_*A*,1_ = 1 and *y*_*A*,1_ = *p*, which is equivalent to an initial random failure on network *A*.

## Results

We next present these theoretical results using several simple examples and verifying them with stochastic simulations.

**Figure 2:**
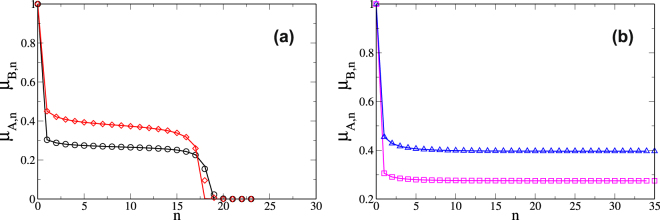
Temporal evolution, close to the critical threshold, of the giant component *μ*_*A*_(*n*) and *μ*_*B*_(*n*) of networks *A* and *B*, when both are random regular (RR) networks with delta degree distribution *P*_*X*_(*k*) = *δ*_*k*,5_, with *X* = *A*,*B*. The degree distributions of supply links are also delta-distributions with *P*_*sA*_(*k*) = *P*_*sB*_(*k*) = *δ*_*k*,5_ and *k*_s_^*^ = 2. The critical threshold for this system is *p*_*c*_ = 0.381. (**a**) *p* = 0.38, (**b**) *p* = 0.381. Network A (○, ), Network B (, ). The dashed lines are the results from the equations and the symbols are the results from the stochastic simulations.

To test the validity of the equations, Fig. 2 shows the temporal evolution of the order parameter of networks *A* and *B* close to the critical threshold *p*_*c*_, computed using the equations and stochastic simulations when the giant component functionality rule is applied (see Supplementary Information: subsections *Giant Component* and *Numerical Solution for the threshold p*_*c*_). Note that the plots show the simulation results are in total agreement with the theoretical results.

**Figure 3:**
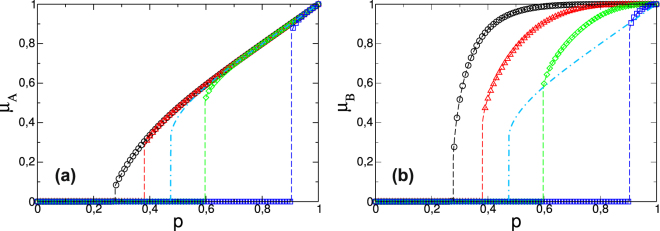
Two random regular (RR) networks with *P*_*A*_(*k*) = *P*_*B*_(*k*) = *δ*_*k*,5_ and *P*_*sA*_(*k*) = *P*_*sB*_(*k*) = *δ*_*k*,5_ and system size *N* = 10^5^ for different values of required supplies, ${k}_{sX}^{\ast }$ = 1 (○), ${k}_{sX}^{\ast }$ = 2 (), ${k}_{sX}^{\ast }$ = 3 (), ${k}_{sX}^{\ast }$ = 4 (), as a function of the initial fraction of survived nodes *p*. Also shown two RR networks with *P*_*A*_(*k*) = *P*_*B*_(*k*) = *δ*_*k*,5_, but *P*_*sA*_(*k*) = *P*_*sB*_(*k*) = *δ*_*k*,1_, ${k}_{sX}^{\ast }$ = 1 (). The symbols are the results of the stochastic simulations and the lines are the iterated values obtained by equations (3–5). The dashed-dotted lines represent only the theoretical results since they have been obtained in Ref.^11^. In panels (a) and (b) we show the order parameter of network *A* and *B*, *μ*_*A*_ and *μ*_*B*_, respectively for the giant component rule.

Figure 3 shows a plot of *μ*_*A*_ and *μ*_*B*_ in the steady state as a function of the initial fraction of surviving nodes *p* when the giant component rule is applied. The results for the k-core rule are shown in the Supplementary Information. We use two random regular (RR) networks with a degree distribution *P*_*X*_(*k*) = *δ*_*k*,5_, with *X* = *A*, *B*, and where the distribution of supplies is also RR with *P*_*s*,*A*_(*k*) = *P*_*s*,*B*_(*k*) = *δ*_*k*,5_. For the external rule of functionality we use *r*_*sX*_(*j*, *k*) = 0 if *j* < *m* and *r*_*sX*_(*j*, *k*) = 1 if *j* ≥ *m* for all *m* from *m* = 1 to *m* = 4. The results obtained from the equations (dashed lines) agree with the results of the simulations (symbols). In addition we compare the results of the present model with the results of the original model of cascading failures^11^ shown as a dashed-dotted line in which *P*_*X*_(*k*) = *δ*_*k*,5_, but *P*_*s*,*A*_(*k*) = *P*_*s*,*B*_(*k*) = *δ*_*k*,1_ and *m* = 1.

Note that in network *A* the order parameter for all values of *k*_s_^*^ is proportional to *p* until it begins to drop and become close to the critical threshold *p*_*c*_. This means that the depletion of the supply from network *B* does not significantly impact network *A* until it reaches the collapse threshold at which the system breaks down with a discontinuous transition. We calculate this critical value numerically using the generating functions (see Supplementary Information: section *Numerical solution for the threshold p*_*c*_). Note also that, as expected, the behavior of network *B* is different. Because there is no initial random failure in network *B*, it remains more intact than network *A*. When network *A* crumbles, however, both networks collapse. Thus despite its damage being minor the transition in network *B* is more abrupt, more unexpected, and, therefore, more dangerous. This is the key difference between the present mode and the original model^11^ in which the behaviors of network A and B are identical. In addition, note that the system is more resilient when *k*_s_^*^ is smaller, i.e., when the supply level decreases. We also observe that the interdependent system with only one supply-demand link (the dashed-dotted line) is more resilient than a system with more connections between the two networks, but with large functionality thresholds *m* ≥ 3.

If instead of the giant component we apply the k-core as an internal functionality rule we get the same qualitative results. For different values of *k* ^*^ and *k*_s_^*^ the order parameters also undergo a discontinuous transition, and the system becomes more vulnerable when the threshold of internal links and the threshold of supply links increases (see Supplementary Information: section *k-core Percolation*).

**Figure 4:**
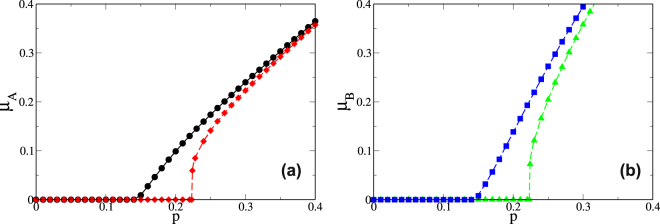
Order parameters for the “mass rule”, for a system of networks with internal distribution *P*_*A*_(*k*) = *P*_*B*_(*k*) = *δ*_*k*,5_, supply distributions *P*_*sA*_(*k*) = *P*_*sB*_(*k*) = *δ*_*k*,2_ and thresholds *k*_A_^*^ = *k*_B_^*^ = 1. All the components of size *h* = 1 are deleted (*q*(1) = 1), and all the components of size *h* ≥ 3 are preserved (*q*(3) = *q*(4) = ... = *q*(*h*_*max*_) = 0 where *h*_*max*_ is the maximum value of *h*). The curves represent the case *q*(2) = 0.3 (●, ), for which there is a continuous transition, and *q*(2) = 0.85 (, ), which leads to an abrupt breakdown of the order parameter. The dashed lines represent the theoretical results and the symbols the stochastic simulations. (**a**) Network *A*, (**b**) Network *B*.

When applying the “mass” rule, finite components of size *h* in network *X* survive with a probability 1 − *q*_*X*_(*h*). When all nodes have a single supply-demand link, i.e., when *k*_*s*_ = 1 and *k*_s_^*^ = 1, and all finite components of size greater than or equal to *h* = 2 are preserved, the system undergoes a continuous transition^28^. Here *q*_*X*_(1) = 1 and *q*_*X*_(*h*) = 0 for *h* ≥ 2. If the number of supply links increases and the threshold *k*_s_^*^ = 1 is fixed, the system becomes more resilient and the transition remains continuous. In contrast, if all the components of size *h* = 2 are removed [*q*_*X*_(2) = 1] the transition becomes discontinuous irrespective of the number of supply-demand links connecting the networks. Nevertheless, not all the components of size *h* = 2 need to survive to have a continuous transition. Figure 4 shows the order parameters for *q*(2) = 0.3 and *q*(2) = 0.85 when *q*_*A*_(*h*) = *q*_*B*_(*h*) = *q*(*h*). Note that when *q*(2) = 0.3 the transition is continuous even when some of the components of size *h* = 2 are deleted. When *q*(2) = 0.85 the number of surviving *h* = 2 components is insufficient to prevent an abrupt transition.

**Figure 5:**
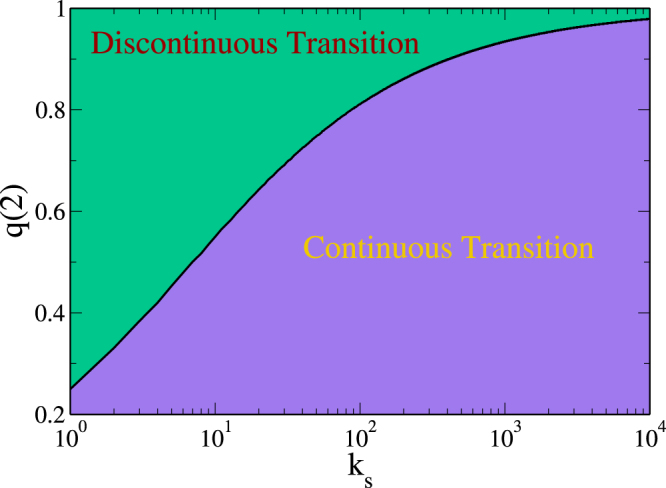
Phase diagram that shows the continuous and discontinuous transitions zones when the “mass rule” is applied. The curve represents the critical probability of failure of the components of size *h* = 2 as a function of the number of the supply-demand links. In this case *P*_*A*_(*k*) = *P*_*B*_(*k*) = *δ*_*k*,5_, *P*_*sA*_(*k*) = *P*_*sB*_(*k*) = *δ*_*k*,*ks*_ and *k*_sA_^*^ = *k*_sB_^*^ = 1. For clarity, the *k*_*s*_ axis is shown on a log scale.

Thus when *k*_s_^*^ = 1 there is a critical value of *q*(2) = *q*_*c*_(2) that separates the zone of continuous transition from the zone of discontinuous transition. Figure 5 shows a phase diagram for a system of networks following the “mass” rule with an internal distribution *P*_*A*_(*k*) = *P*_*B*_(*k*) = *δ*_*k*,5_ and supply distribution *P*_*sA*_(*k*) = *P*_*sB*_(*k*) = *δ*_*k*,*ks*_. Note that the behavior of the critical probability as a function of the number of supply-links *k*_*s*_ between the networks delimits these two zones. As *k*_*s*_ increases the system becomes more robust, and more components must fail to cause an abrupt transition. In the limiting case *k*_*s*_ → ∞ the curve reaches the value *q*_*c*_(2) = 1, but also *p*_*c*_ → 0. On the other hand, when *k*_s_^*^ > 1 the transition is always discontinuous for any value of *q*(*s*) and sufficiently large *k*_*s*_.

**Figure 6:**
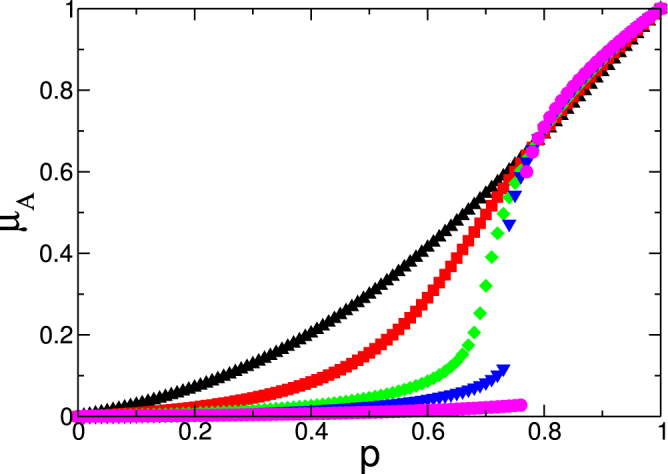
Order parameter of network *A* as a function of the initial failure for a bipartite system and for a threshold function *r*_*sX*_(*j*, *k*_*sX*_) = 3(*j*/*k*_*sX*_)^2^ − 2(*j*/*k*_*sX*_)^3^. The supply-demand distribution is single valued with *k*_*sX*_ = 3 (▲), *k*_*sX*_ = 5 (), *k*_*sX*_ = 7 (), *k*_*sX*_ = 8 () and *k*_*sX*_ = 10 (). For *k*_*s*_ ≥ 8 there is a discontinuous transition. The curves were obtained from the equations.

What happens if no internal functionality rule is applied? This could be the case in a bipartite system in which nodes within each network do not interact but use nodes in the other network as bridges to establish connections. Here the exacerbation factor is simply *g*_*X*_(*y*) = 1, which simplifies the equations. If we analyze this system for different functions *r*_*sX*_(*j*,*k*) (see Supplementary Information: section *Examples of r*_*sX*_(*j*,*k*) *functions*) we see that if *r*_*sX*_(*j*,*k*) is a step function with fixed threshold ${k}_{sX}^{\ast }$ = 2, the transition is continuous, but it is discontinuous for ${k}_{sX}^{\ast }$ > 2, and there is no transition for *p* > 0 if ${k}_{sX}^{\ast }$ = 1. Also if we choose a linear function, i.e., *r*_*sX*_(*j*, *k*_*sX*_) = *j*/*k*_*sX*_, there is again no transition because here functions *W*_*s*_(*β*) and *Z*_*s*_(*β*) become linear functions of *β*. On the other hand, when the function *r*_*sX*_ is by nonlinear, the behavior changes. Figure 6 shows the behavior of the order parameter of network *A* for a polynomial function *r*_*sX*_(*j*, *k*_*sX*_) = 3(*j*/*k*_*sX*_)^2^ − 2(*j*/*k*_*sX*_)^3^ and for a supply-demand distribution *P*_*s*,*X*_(*k*) = *δ*_*k*,*ks*_. Note that for small values of *k*_*s*_ the order parameter moves smoothly to zero but for *k*_*s*_ = 8 the system undergoes a discontinuous transition. The existence of these transitions can be explained studying Eqs (3) and (4) (see Supplementary Information: section *Numerical solution for the threshold p*_*c*_).

Unlike the previous results, the transition here does not produce a total collapse of the system, and after the jump a small fraction of nodes remains functional for any *p* > 0. If a delta-distribution of supply links is replaced by the Poisson distribution with 〈*k*_*s*_〉_*X*_ = *λ*, we find a critical point on a (*p*, *λ*) plane *λ*_*c*_ = 7.58465, *p*_*c*_ = 0.728102 at which the first order phase transition emerges. For *λ* > *λ*_*c*_ the transition is first order and for *λ* < *λ*_*c*_ there is no phase transition for *p* > 0. At this point the system belongs to the mean-field universality class, such as the Ising model in infinite dimensions where *p* corresponds to the ordering field and *λ* to the thermal field.

We next analyze the limiting case of large *k*_*s*_ values when all nodes in network *B* have a fixed threshold *k*_sB_^*^, and we find that the collapse threshold *p*_*c*_ converges to a value determined by the ratio *γ* ≡ *k*_sB_^*^/*k*_*sB*_ given by

6 $$\gamma ={p}_{c}{g}_{A}({p}_{c}),$$

which is valid for all of the internal failure rules.

The *p*_*c*_ value depends on *γ* in this limit because when 〈*k*_*sX*_〉 → ∞ the functions *W*_*sB*_(*β*) and *Z*_*sB*_(*β*) become step functions equal to 0 for *β* < *γ* and to 1, otherwise. Note that *γ* only relates to the external properties of network *B*, but that the value of *p*_*c*_ depends solely on the topology of network *A*. This is because network *B* is intact above *p*_*c*_, but when *p* < *p* *c* all the supply-demand links maintaining the integrity of network *B* fail and the entire structure crumbles. Thus here the topology of network *B* does not affect the final state of the system. See Supplementary Information: section *Asymptotic properties of the functions W*_*s*_ *and Z*_*s*_ for the derivation of Eq. (6).

**Figure 7:**
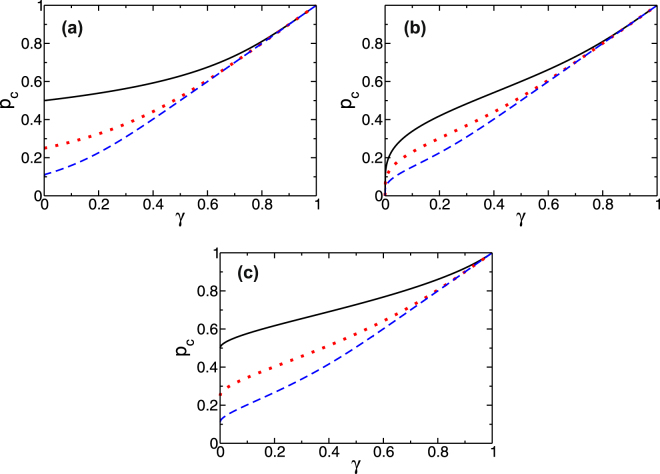
Critical threshold *p*_*c*_ as a function of *γ* = *k*_sB_^*^/*k*_*sB*_ for different values of *z*_*A*_, the internal connectivity of network *A*, where its internal degree distribution is RR. The curves represent different values of *z*_*A*_: *z*_*A*_ = 3 (**─**), *z*_*A*_ = 5 () and *z*_*A*_ = 10(). Panel (a) corresponds to the Giant Component rule. Panel (b) corresponds to the “mass rule”, with *q*(*h*) = 1 for *h* = 1,2,3, and panel (c) to the k-core rule with *k*_X_^*^ = 2 Note that in panel (b) *p*_*c*_ ~*γ* ^1/4^ when *γ* → 0, and thus corresponding curves appear finite even for very small *γ* > 0.

Figure 7 shows the behavior of Eq. (6) for each internal rule of functionality and for several values of internal connectivity *z*_*A*_ in network *A* when it has an internal degree distribution *P*_*A*_(*k*) = *δ*_*k*,*zA*_. Note that all curves go to *p*_*c*_ = 1 when *γ* → 1, i.e., ${k}_{sB}^{\ast }\sim {k}_{sB}$, and thus even a small perturbation can cause a system breakdown. In contrast, curves with higher *z*_*A*_ values have lower *p*_*c*_ values because increased connectivity means increased resilience. In addition, when *γ* → 0 then ${k}_{sB}\gg {k}_{sB}^{\ast }$, rendering the influence of network *B* on network *A* insignificant. Here network *A* behaves as an isolated system. We see this in the giant component rule [see Fig. 7a] in which *p*_*c*_ → 1/(*z*_*A*_ − 1) as *γ* → 0, a value that corresponds to the critical threshold of node percolation^43,44^ in isolated RR networks. Similarly, for the “mass” rule we find that *p*_*c*_ → 0 when *γ* → 0 because when there is an initial attack 1 − *p* on an isolated network there are always components of varying masses in the thermodynamic limit (with an infinite number of nodes). Thus for any size *h* there are always surviving components when *p* > 0.

If there is a Poisson internal degree distribution in network *A*, i.e., *P*_*A*_(*k*) = *exp*[−〈*k*〉_*A*_]〈*k*〉_*A*_^*k*^/*k*! where 〈*k*〉_*A*_ is the mean connectivity, we can write a closed-form expression for *p*_*c*_ for the giant component rule,

7 $${p}_{c}=\frac{\gamma }{1-{\exp }[-\gamma \,{\langle k\rangle }_{A}]}\mathrm{.}$$

Note that *p*_*c*_ does not depend on the internal degree distribution of network *B*. The derivation of Eq. (7) is supplied in the Supplementary Information: section *Asymptotic properties of the functions W*_*s*_ *and Z*_*s*_. On the other hand, if the system is bipartite then from Eq. (6) the critical value is simply *p*_*c*_ = *γ*.

## Discussion

We have analyzed the cascading failure process in a system of two interdependent networks in which nodes within each network have multiple connections, or supply-demand links, with nodes from their counterpart network. In this model each node must have at least a given number of supply-links leading to functional nodes in the other network to remain active. We call this number the supply threshold and we call this condition the external functionality rule. We have studied the process under three internal functionality rules, (I) nodes must belong to the giant component in their own network, (II) nodes that belong to a finite component survive with a probability determined by the mass of the component, and (III) an internal version of the external functionality rule, known as heterogeneous k-core percolation. In addition, we have studied a system in the absence of any internal functionality rule, which is equivalent to a bipartite network. Our system is a generalization of the models of interdependent networks^11,13^ that represent a particular case of our model with *P*_*sX*_(*k*) = 0 for *k* > 1 and a giant component rule of internal functionality. Our model shows a rich behavior for various parameter values that is characterized by the appearance of discontinuous first order transitions. In some cases, multiple first order transitions can be observed, a situation impossible in the original models^11,13^.

We have found that for all the internal functionality rules the system is more robust when the supply threshold is lower. Under internal rules I and III there is a discontinuous transition at a collapse threshold *p* = *p*_*c*_. The main difference between our model and the previously studied models^11,13^ is that in the case of multiple supply links the initial attack on network *A* does not immediately affect network *B*, and it remains more functional than network *A* for any *p* > *p*_*c*_. This makes the transition, when it occurs in network *B*, more abrupt than in network *A*. These sudden breakdowns can come without warning. In some catastrophic events, e.g., an earthquake of sub-threshold strength, the damage to network *B* may be minor and the development of precautions or recovery strategies thus deemed of minor importance. This becomes problematic when the strength of an earthquake exceeds a certain threshold and causes a total breakdown in network *B*. In contrast, in “mass” rule II for *k*_s_^*^ = 1 the transition can be continuous depending on the probability that components of size *h* = 2 remain functional and on the number of supply-demand links. For each value of *k*_*s*_ there is a critical probability *q*(2) below which the transition becomes discontinuous.

When the model is applied to a bipartite system, the behavior is determined by function *r*_*sX*_. In particular, when this function is polynomial there is no transition in *k*_*sX*_ ≤ 7, but when *k*_*s*_ increases this curve breaks and becomes discontinuous.

Finally we have studied the asymptotic limit value of the number of supply-demand links, and find that when *r*_*sB*_ is a step function there is an exact relationship between the ratio *γ* = k_sB_^*^/*k*_*sB*_ and the collapse threshold *p*_*c*_. We also find that in this limit the resilience of the interacting system is enhanced up to the point at which the critical threshold *p*_*c*_ is solely dependent on the topology of network *A*.

## Methods

For the stochastic simulations we use for both networks a system size of *N* = 10^6^ to compute the steady state and *N* = 10^8^ for the temporal evolution close to the critical threshold (See Fig. 2). We use the Molloy-Reed Algorithm^45^ for the construction of the networks. The simulation results are averaged over 1000 network realizations.

For model II, the “mass” rule, a finite component of size *h* survives with probability 1 − *q*(*h*). In the stochastic simulations if a finite component remains after the internal failure at a step of the cascade, then in the following steps of the cascade this component only can fail due to the external rule of functionality.

In our theoretical analysis, to calculate the values of the order parameters at the steady state we iterate the temporal evolution Eqs (3)–(5) until the condition *μ*_*A*_ ≡ *μ*_*A*,*n*_ = *μ*_*A*,*n*__− 1_ is satisfied. At this stage the magnitudes of all order parameters reach a steady state and no longer change.

## Electronic supplementary material

Supplementary Information
