# Supplementary material for: Cascading Failures in Interdependent Networks with Multiple Supply-Demand Links and Functionality Thresholds
Source: Sci Rep. 2017 Nov 8;7:15059. doi: 10.1038/s41598-017-14384-y (PMC5678122; doi:10.1038/s41598-017-14384-y)
Supplement: Supplementary file 1 — Supplementary Information [file 41598_2017_14384_MOESM1_ESM.pdf]

Cascading Failures in Interdependent Networks with Multiple Supply-Demand Links and Functionality Thresholds
Supplementary Information

M. A. Di Muro[1,*], L. D. Valdez[2,3], H. H. Aragão Rêgo[4], S. V. Buldyrev[5], H.E. Stanley[6], and L. A. Braunstein[1,6]

[1]Instituto de Investigaciones Físicas de Mar del Plata (IFIMAR)-Departamento de Física, Facultad de Ciencias Exactas y Naturales, Universidad Nacional de Mar del Plata-CONICET, Funes 3350, (7600) Mar del Plata, Argentina.
[2]Instituto de Física Enrique Gaviola, CONICET, Ciudad Universitaria, 5000 Córdoba, Argentina.
[3]Facultad de Matemática, Astronomía, Física y Computación, Universidad Nacional de Córdoba, Córdoba, Argentina
[4]Departamento de Física, Instituto Federal de Educação, Ciência e Tecnologia do Maranhão, São Luís, MA, 65030-005, Brazil
[5]Department of Physics, Yeshiva University, 500 West 185th Street, New York, New York 10033, USA
[6]Center for Polymer Studies, Boston University, Boston, Massachusetts 02215, USA
[*]mdimuro@mdp.edu.ar

Explicit form of the functionality rules

Giant component

The giant component in a network is the largest connected component. Most functioning networks are completely connected, but when they experience failure, finite components—little islands of nodes—become disconnected from the giant component. A common functionality rule states that nodes in these finite components have insufficient support to remain active. Thus in addition to the nodes rendered inactive by failure, the exacerbation factor renders inactive all nodes not connected to the giant component. If network X has a degree distribution $P_X(k)$ and a fraction $1-y_X$ of nodes is randomly removed, the exacerbation factor g_X is $g_X(y_X) = 1 - G_0^X[1-y_X(1-f_\infty^X)]$, where f_∞^X is the probability that the branches do not expand to infinity, and it satisfies the recurrent equation $f_\infty^X = G_1^X[1-y_X(1-f_\infty^X)]$. The functions $G_0^X(u)$ and $G_1^X(u)$ are the generating functions of the degree distribution and the excess degree distribution, respectively. They are given by $G_0^X(u) = \sum_k P_X(k)u^k$ and $G_1^X(u) = \sum_k k/\langle k\rangle_X P_X(k)u^{k-1}$, where $\langle k\rangle_X$ is the average connectivity of network X, $\langle k\rangle_X = \sum_k k\, P_X(k)$.

Finite components

We can relax the giant component rule and allow some finite components to be self-sustaining and remain functional. If we allow the giant component to remain active after a failure and also some of the finite components to remain active with a probability related with their size h, then the exacerbation factor is

$$g_X(y_X) = 1 - \sum_h q_X(h)\pi_{h,X}(y_X), \tag{1}$$

where $q_X(h)$ the probability that a component of size h has been removed, and $\pi_{h,X}(p)$ the probability that a randomly-selected surviving node belongs to a component of size h. We can obtain the functions

$\pi_{h,X}(y_X)$ using the Lagrange inversion formula[1] for any given distribution $P_X(k)$.

k-core Percolation

In conventional or homogeneous k-core percolation, every node has an identical threshold k^*. Thus following a failure, if the number of surviving nodes among the k neighbors of a node is less than k^*, the node fails, otherwise it remains functional. In contrast, in heterogeneous k-core percolation each node i with initial degree k_i has a randomly assigned threshold $k_i^* \leq k_i$. In heterogeneous k-core percolation, the distribution of thresholds k_i^* is given by the cumulative distribution $r_X(j,k) = P(k^* \leq j|k)$, where k denotes the degree values of network nodes. The simplest $r_X(j,k)$ case is a step function, i.e., $r_X(j,k) = 0$ if $j < k^*$ and $r_X(j,k) = 1$ if $j \geq k^*$ and for all k. This is equivalent to assigning all nodes the threshold $k_i^* = k^*$, which is equivalent to homogeneous k-core percolation. Another option is the linear function $r_X(j,k) = k^*/k$ in which the thresholds k^* for nodes with an initial degree k are uniformly distributed between 1 and k.

If we know the degree distribution $P_X(k)$ and the threshold distribution $r(j,k)$, we can define the heterogeneous k-core generating function

$$W_X(\beta) = \sum_{k=0}^{\infty} P_X(k) \sum_{j=0}^{k} \binom{k}{j} r_X(j,k) \beta^j (1-\beta)^{k-j}, \tag{2}$$

and the k-core generating function of the excess distribution,

$$Z_X(\beta) = \sum_{k=1}^{\infty} \frac{kP_X(k)}{\langle k \rangle_X} \sum_{j=0}^{k-1} \binom{k-1}{j} r_X(j+1,k) \beta^j (1-\beta)^{k-j-1}. \tag{3}$$

The exacerbation factor of the k-core heterogeneous percolation can thus be written $g_X(y_X) = W_X(\beta)$, where as in Sec. 1.1 β satisfies the self-consistent equation $\beta = y_X Z_X(\beta)$, and y_X is the fraction of surviving nodes in network X.

Figure 1 plots μ_A and μ_B in the steady state for the k-core rule.

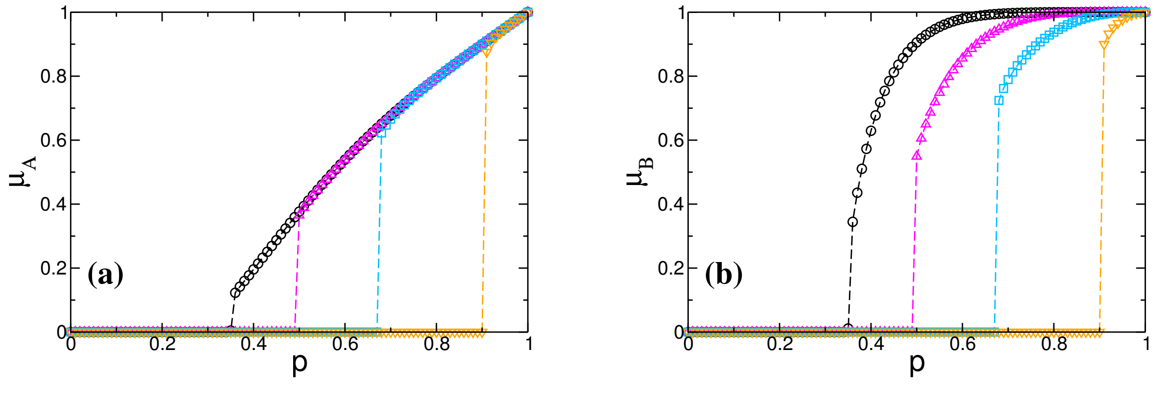

Figure 1. Order parameters for the homogeneous k-core rule with $k^* = 2$ as a function of the initial fraction of survived nodes p, for two random regular (RR) networks with $P_{sA}(k) = P_{sB}(k) = \delta_{k,5}$ and system size $N = 10^5$, and different values of required supplies, $k_s^* = 1$ (○), $k_s^* = 2$ (△), $k_s^* = 3$ (□) $k_s^* = 4$ (▽). The symbols are the results of the stochastic simulations and the lines the iterated values from the equations. (a) Network A. (b) Network B.

Numerical Solution for the threshold p_c

The critical point p_c at which the transition takes place can be determined using the equations from the main text. We combine the equations in set (3) at the steady state at which $f_{X,n} = f_{X,n-1} = f_X$, thus withdraw the f_B-dependence, and obtain an equation in terms of f_A,

$$f_A = F(f_A) \equiv Z_{sB}\left[\eta(f_A)\right] g_B\left[W_{sB}[\eta(f_A)]\right], \tag{4}$$

with $\eta(f_A) \equiv f_B = pZ_{sA}(f_A) g_A\left[pW_{sA}(f_A)\right]$.

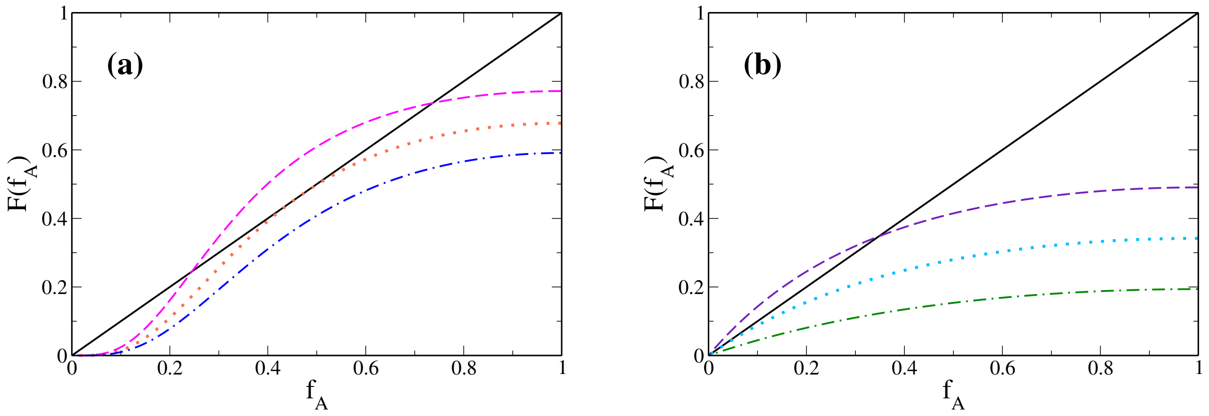

Figure 2. Graphical solution of Eq. (4) for a system of two RR networks with $z = 5$, $P_{sX}(k) = \delta_{k,2}$, in which all nodes have the same threshold $k_s^* = 1$. In this case we use the "finite components" rule, with $q_X(1) = 1$ and $q_X(h) = 0$ for $h > 2$. In (a) we have $q_X(2) = 1$, which results in a discontinuous transition. The curves represent different values of p: $p = 0.26$ (- - -), $p = 0.22$ (-·-·) and $p = p_c = 0.2374$ (·····). For the critical threshold, the curve is tangent to the identity at the solution $f_A > 0$. In (b) we show a continuous transition with $q_X(2) = 0$, in which $p = 0.13$ (- - -), $p = 0.07$ (-·-·) and $p = p_c = 0.1$ (·····). For the curve that represents the critical value, the point of tangency is located at $f_A = 0$.

For a given value of p, the solution is the intersection between function F and the identity. Above p_c there is always a non-trivial solution. When $p < p_c$, Eq. (4) is only valid for $f_A = 0$. The method of finding the critical point differs depending on whether the transition is discontinuous or continuous. Figure 2 shows the graphical solution of Eq. (4) for (a) a discontinuous transition and (b) a continuous transition. We plot the curves for $p = p_c$, $p > p_c$, and $p < p_c$. When the transition is abrupt, when $p = p_c$ function F is tangent to the identity at $f_A = f_{Ac}$, which is the solution to Eq. (4) for the critical threshold. Thus we have a condition that must be fulfilled at the critical point,

$$\frac{dF(f_A)}{df_A} = 1. \tag{5}$$

Thus we can solve Eqs. (4) and (5) numerically to find the critical threshold p_c for a discontinuous abrupt transition.

In contrast, Fig. 2(b) shows that function F is also tangent to the identity, but here at $f_A = 0$. Thus for a continuous transition we can find the critical value p_c by solving Eq. (5) when $f_A = 0$.

For the mass rule, the type of transition is related to the survival probability of the $h = 2$ components. If we assume here that all the $h = 1$ components and a fraction $q(2)$ of the $h = 2$ components fail, then for single-value internal degree distributions and supply distributions when $k_s^* = 1$, the derivative of function F at the critical threshold evaluated at the origin is

$$\left.\frac{dF(f_A)}{df_A}\right|_{f_A=0} = \Big(p_c\, k_s\, k\, [1-q(2)]\Big)^2. \tag{6}$$

Thus when $q(2) = 1$ the continuity condition is fulfilled only for $k_s \to \infty$, as we can see in Fig. 5 in the main text.

On the other hand we can use Eq. (4) to understand the transitions present in Fig. 6 from the main text. Here the system is bipartite and thus we apply no internal functionality rule. The system also has a supply distribution $P_s(k) = \delta_{k,k_s}$ and a supply threshold distribution $r_{sX}(j,k) = 3(j/k)^2 - 2(j/k)^3$.

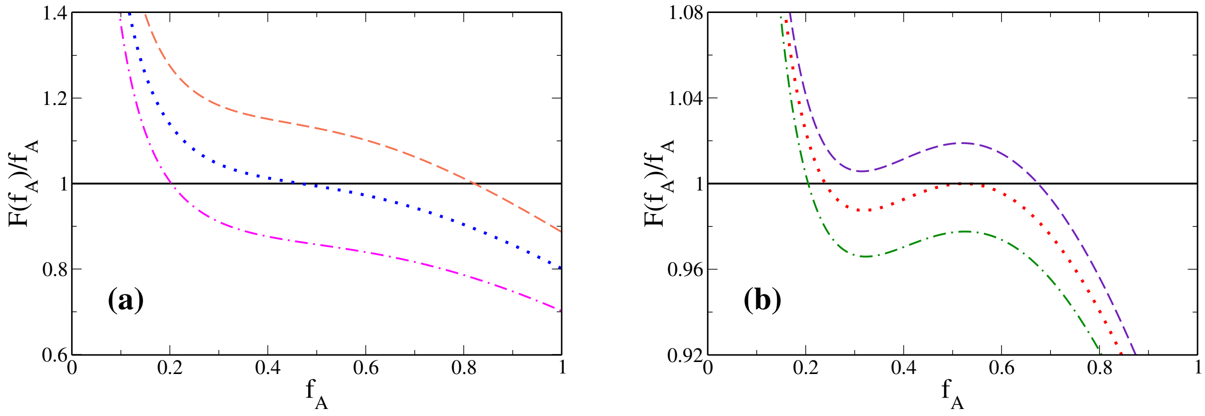

Figure 3. Graphical solution of Eq. (4) for a bipartite system with a supply distribution $P_s(k) = \delta_{k,k_s}$, and a supply threshold distribution $r_{sX}(j,k) = 3(j/k)^2 - 2(j/k)^3$. Unlike Fig. 2 here the y axis is divided by the x axis to have a better visualization of the intersection points. In (a) we have $k_{sX} = 7$, value for which there is a continuous transition for $p = 0$, as for $p > 0$ the curve always intersect the identity at a non-zero point. The curves represent $p = 0.6$ (-·-·), $p = 0.7$ (·····) and $p = 0.8$ (- - -) . On the contrary in (b) we have $k_{sX} = 8$ and there is a discontinuous transition. For $p = 0.75$ (- - -) there is only one point of intersection, but for lower values of p two more solutions appear, although the largest of them is the one related with the process. For $p = 0.73626$ (·····) two of the solutions fuse together, and the curve becomes tangent to the identity, thus the solution for this value of p is the point of tangency. However for lower values of p the point of intersection of the left is the only solution of the process, as we can observe for $p = 0.72$. (-·-·)

Figure 3 shows the graphical solution for this system for different values of initial failure p. Figure 3(a) shows $k_s = 7$. Note that the curve always intersects the identity at a non-zero value when $p > 0$. Thus the order parameter goes to zero in a continuous transition when $p = 0$. In contrast, Fig. 3(b) shows $k_s = 8$, and the curve behaves differently. For large values of p there is only one solution, but decreasing p produces a new lower stable solution. A point of intersection is a stable solution when a iterative process converges to this point. If initially $f_A = 1$, i.e., network B is intact at the beginning of the cascade,[3] then successive iterations of Eq. (4) converge to the highest solution. However when p further decreases there is a critical value at which the largest solution suddenly vanishes, and the iterative process converges to

the lower point, which is now the only solution. This abrupt change of solutions causes the discontinuous transition shown in Fig. (6) in the main text.

Asymptotic properties of the functions W_s and Z_s

We next study the case in which r_{sX} is a step function $\Theta(k_s - k_s^*)$, i.e., when all nodes have the same threshold k_s^*. Using the supply distribution $P_{sX}(k) = \delta_{k,k_s}$ for simplicity, we show that the behavior of the order parameters does not depend on the values of k_s^* and k_s when k_s is large, but on the ratio $k_s^*/k_s \equiv \gamma$. We assume that networks A and B have the same external properties, and we drop index X. Here we rewrite function $W_s(x)$ [see Eq. (1) from the main text] using the incomplete beta function

$$W_s(x) = 1 - (k_s - k_s^* + 1)\binom{k_s}{k_s^* - 1}\int_0^{1-x} t^{k_s - k_s^*}(1-t)^{k_s^* - 1}\, dt, \tag{7}$$

and it is thus straightforward to compute the first derivative of W_s

$$W_s^{'}(x) = (k_s - k_s^* + 1)\binom{k_s}{k_s^* - 1} x^{k_s^* - 1}(1-x)^{k_s - k_s^*}. \tag{8}$$

.

When W_s converges to a step function for large k_s its derivative $W_s^{'}$ converges to a Dirac delta centered on x_c. To verify this we calculate the mean of this function and its variance,

$$\langle x \rangle \;=\; \frac{k_s^*}{k_s + 1} \tag{9}$$

$$\sigma_x \;=\; \frac{k_s^*(k_s^* + 1)k_s!}{(k_s + 2)!} - \left(\frac{k_s^*}{k_s + 1}\right)^2.$$

For a fixed value $\gamma \equiv k_s^*/k_s$ the variance goes to zero when $k_s \to \infty$, indicating that function W_s is discontinuous at $x = x_c$. The previous analysis is also valid for Z_s, but when $k_s^* > 1$, since $Z_s(x) = 1$ when $k_s^* = 1$.

Thus $W_s(x)$ and $Z_s(x)$ (when $k_s^* > 1$) converge to a Heaviside distribution, which depends on the ratio γ for $k_s \to \infty$,

$$W_s(x) = Z_s(x) = \begin{cases} 0 & x < \gamma; \\ \frac{1}{2} & x = \gamma, . \\ 1 & x > \gamma. \end{cases} \tag{10}$$

Then in this limit the solution of Eqs. (1)–(3) in the steady state and the transition point p_c is dependent only on γ.

If we know the asymptotic properties of these functions, we can determine the critical point p_c behavior at this limit. As $p \to p_c$ we expect that $f_B < f_A$, since network A receives the initial failure, i.e., the probability f_B that an external link from network A to B leads to a functional node in network A is lower than in the opposite direction. Then using Eq. (10) at criticality $Z(f_B) = W(f_B) = 0.5$ and $Z(f_A) = W(f_A) = 1$, i.e., $f_A > \gamma$ and[4] $f_B = \gamma$. Then the set (1) equations in the main text in the steady state at $p = p_c$ can be rewritten

$$f_A \;=\; \frac{1}{2} g_B\left(\frac{1}{2}\right), \tag{11}$$

$$f_B \;=\; \gamma = p_c Z(f_A) g_A\left[p_c W(f_A)\right]. \tag{12}$$

Because $f_A > \gamma$, $Z(f_A) = 1$ according to Eq. (10). Thus from Eq. (S12)

$$\gamma = p_c g_A(p_c), \tag{13}$$

which is related to the value of p_c with γ.

Note that when $k_s^* = 1$, Eq. (S13) becomes $f_A = g_B\left(\frac{1}{2}\right)$. Nevertheless here Eq. (13) still holds.

Using the giant component rule at this limit we find analytically the value of the criticality threshold for a particular case. If network A has an internal Poisson degree distribution, i.e., if $P_A(k) = \langle k \rangle_A^k exp[-\langle k \rangle_A]/k!$ where $\langle k \rangle_A$ is the average internal connectivity of network A, then $G_0^A(x) = G_1^A(x) = exp[\langle k \rangle_A(x-1)]$. Here $\gamma = p_c(1 - f_\infty^A)$, and thus we obtain

$$p_c = \frac{\gamma}{1 - exp[-\gamma \langle k \rangle_A]}. \tag{14}$$

Examples of $r_s(j,k)$ functions

Equations

$$W_{sX}(f) = \sum_{k=0}^{\infty} P_{sX}(k) \sum_{j=0}^{k} r_{sX}(j,k) C_{k,j} f^j (1-f)^{k-j}. \tag{15}$$

and

$$Z_{sX}(f) = \sum_{k=0}^{\infty} \frac{k P_{sX}(k)}{\langle k_s \rangle_X} \sum_{j=0}^{k-1} r_{sX}(j+1,k) C_{k-1,j} f^j (1-f)^{k-j-1}, \tag{16}$$

can be evaluated explicitly for the power law shape

$$r_{sX}(j,k) = \left(\frac{j}{k}\right)^m, \tag{17}$$

when $k > 0$ and $r_{sX}(0,0) = 1$. The latter condition is to prevent autonomous nodes with no supply links from dying. Obviously this shape can be generalized to any polynomial by which any function $r_{sX}(j,k)$ can be approximated. Successively applying operator fd/df to the corresponding probability generating function, i.e., binomials $(f+q)^k$, and letting $q = 1-f$ allows us to express functions $W_{sX}(f)$ and $Z_{sX}(f)$ as polynomials of power m of f

$$W_{sX}(f) = \sum_{n=0}^{m} w_n^m f^n + P_{sX}(0) \qquad Z_{sX}(f) = \sum_{n=0}^{m} z_n^m f^n, \tag{18}$$

with coefficients that can be expressed through negative moments of the distribution $P_{sX}(k)$

$$w_n^m = S(m,n) \sum_{k=1}^{\infty} P_{sX}(k) \frac{k!}{k^m (k-n)!} \tag{19}$$

and

$$z_n^m = \frac{S(m+1,n+1)}{\langle k_s \rangle_X} \sum_{k=1}^{\infty} P_{sX}(k) \frac{k!}{k^m (k-1-n)!}, \tag{20}$$

where $S(m,n)$ are Stirling numbers of the second kind that obey recursion relation $S(m+1,n) = nS(m,n) + S(m,n-1)$ with initial conditions $S(0,0) = 1$, $S(0,n)$=0 when $n > 0$, and $S(m,0) = 0$ when $m > 0$.

For linear r_{sX} ($m = 1$), functions $W_{sX}(f)$ and $Z_{sX}(f)$ are linear functions of f

$$W_{sX}(f) = P_{sX}(0) + \Big(1 - P_{sX}(0)\Big) f \qquad Z_{sX}(f) = \left(1 - \frac{1 - P_{sX}(0)}{\langle k_s \rangle_X}\right) f + \frac{1 - P_{sX}(0)}{\langle k_s \rangle_X}. \tag{21}$$

In the case described in Ref.,[2] $P_{sX}(k) = \delta_{1k}$ produces $W_{sX}(f) = f$ and $Z_{sX}(f) = 1$, and hence recursive Eqs. (4) and (5) from the main text reduce to Eq. (1) in Ref.[2] Even when $P_{sX}(k) \neq \delta_{1k}$, linear r_{sX} leads to the same phenomenon described in Ref.,[2] i.e., there is a first order phase transition when $P_{sX}(0)$, the fraction of the autonomous nodes is small, and there is a second order phase transition when $P_{sX}(0)$ is large.

On the other hand, there are differences in a bipartite system of two networks in which the exacerbation factor is $g_X(y) = 1$. Here linear r_{sX} does not produce a non-trivial phase transition when $p > 0$ because Eqs. (15) and (16) become linear equations of f, but threshold r_{sX} is fixed it produces a first order phase transition. The same is true for a fractional threshold: $r_{sX}(j,k) = 0$ for $j < k\alpha$; $r_{sX}(k,j) = 1$ for $j \geq k\alpha$, where $\alpha \in (0,1)$. We can construct a continuous approximation for the fractional threshold $\alpha = 1/2$ as $r_{sX}(j,k) = 3(j/k)^2 - 2(j/k)^3$. For this r_{sX} and for a single valued distribution $P_{sX}(k) = \delta_{k\ell}$ the smallest value of ℓ for which there is a first order phase transition is $\ell = 8$.

References

1. Di Muro, M. A., Buldyrev, S. V., Stanley, H. E. & Braunstein, L. A. Cascading failures in interdependent networks with finite functional components. *Phys. Rev. E* **94**, 042304 (2016).

2. Buldyrev, S. V., Parshani, R., Paul, G., Stanley, H. E. & Havlin, S. Catastrophic cascade of failures in interdependent networks. *Nature* **464**, 1025–1028 (2010).

3. Recall that f_A is the probability of randomly selecting a supply-demand link that leads to a functional node in network B

4. Note that when $Z_s(f_B) = 0$ the interdependent network collapses, but when $Z_s(f_B) = 1$ the functional network is in the steady state.
